# In Vitro Effects of Photon Beam and Carbon Ion Radiotherapy on the Perineural Invasion of Two Cell Lines of Neurotropic Tumours

**DOI:** 10.3390/life13030794

**Published:** 2023-03-15

**Authors:** Alexandra Charalampopoulou, Amelia Barcellini, Giuseppe Emanuele Frittitta, Giorgia Fulgini, Giovanni Battista Ivaldi, Giuseppe Magro, Marco Liotta, Ester Orlandi, Marco Giuseppe Pullia, Paola Tabarelli de Fatis, Angelica Facoetti

**Affiliations:** 1 Radiobiology Unit, Research and Development Department, CNAO National Center for Oncological Hadrontherapy, 27100 Pavia, Italy; 2 Hadron Academy PhD Course, Istituto Universitario di STUDI Superiori (IUSS), 27100 Pavia, Italy; 3 Radiation Oncology Unit, Clinical Department, CNAO National Center for Oncological Hadrontherapy, 27100 Pavia, Italy; 4 Department of Internal Medicine and Medical Therapy, University of Pavia, 27100 Pavia, Italy; 5 Biology and Biotechnology Department, University of Pavia, 27100 Pavia, Italy; 6 Radiation Oncology Department, Istituti Clinici Scientifici Maugeri IRCCS, 27100 Pavia, Italy; 7 Medical Physics Unit, Clinical Department, CNAO National Center for Oncological Hadrontherapy, 27100 Pavia, Italy; 8 Medical Physics Unit, Istituti Clinici Scientifici Maugeri IRCCS, 27100 Pavia, Italy; 9 Physics Unit, Research and Development Department, CNAO National Center for Oncological Hadrontherapy, 27100 Pavia, Italy

**Keywords:** neurotropism, perineural invasion, migration, radiobiology, NT-3, carbon ions, hadrontherapy

## Abstract

Primary mucosal melanoma (PMM) and pancreatic ductal adenocarcinoma (PDAC) are two aggressive malignancies, characterized by intrinsic radio-chemoresistance and neurotropism, a histological feature resulting in frequent perineural invasion (PNI), supported by neurotrophic factors secreted in the tumour microenvironment (TME), such as neurotrophin-3 (NT-3). Carbon-ion radiotherapy (CIRT) could represent an effective option in unresectable PMM and PDAC. Only a few data about the effects of CIRT on PNI in relation to NT-3 are available in the literature, despite the numerous pieces of evidence revealing the peculiar effects of this type of radiation on tumour cell migration. This in vitro study investigated for the first time the response of PMM and PDAC cells to NT-3 and evaluated the effects of conventional photon beam radiotherapy (XRT) and CIRT on cell viability, proliferation, and migration. Our results demonstrated the greater capacity of C-ions to generally decrease cell viability, proliferation, and migration, while the addition of NT-3 after both types of irradiation determined an increase in these features, maintaining a dose-dependent trend and acting more effectively as a chemoattractant than inductor in the case of migration.

## 1. Introduction

The interaction of cancer cells with diverse cell types in the tumour stroma is known to contribute to the progression and outcomes of human cancers. Despite the well-described interactions of cancer cells with several stromal components, including inflammatory cells, cancer-associated fibroblasts, endothelial cells, and pericytes [1], the investigation of their peculiar relationships with neural cells is still in its early stages. Pancreatic cancer, one of the most lethal cancers, with its abundant stroma, represents one of the best-studied examples of a malignant tumour with a mutually trophic interaction between cancer cells and the intratumoral nerves embedded in the desmoplastic stroma [2]. Although neurotropism is a common feature in melanoma, mostly presented in desmoplastic tumours, it may also be encountered in other forms, such as mucosal melanomas. Since it tends to invade deeply at the primary site and has a high potential to have a positive pathological margin after surgical resection, the presence of neurotropism is believed to be associated with an increased risk of local recurrence and a worse prognosis in cutaneous [3], as well as in mucosal, melanomas of the urogenital tract [4]. Since neurotropism has been investigated in only a few studies regarding its prognostic value in mucosal melanoma (PMM) [5,6] and pancreatic cancer (PDAC) [7], its clinical significance remains largely unknown [8]. Perineural invasion (PNI), defined as the ability of tumour cells to spread via the perineurium space of local peripheral nerves, is a largely forgotten route of metastasis used by solid tumours to spread, together with the other better known routes, such as direct invasion of surrounding tissues, lymphatic spread, haematogenous spread, and seeding along body cavities [9]. PNI is closely associated with increased postoperative locoregional recurrence and a decreased survival rate in several solid tumours, likely because neoplastic cells disseminating along nerve fascicles are spared by macroscopic resection surgery [10,11]. 

Recent studies have revealed that cancer cells have an ability to actively migrate along nerves in a mechanism called neural tracking, which is supported by various molecules, including nerve growth factor (NGF), glial cell line-derived neurotrophic factor (GDNF), neural cell adhesion molecule, matrix metalloproteinases (MMPs), and chemokines, which are secreted by tumour cells and other non-tumour cells in the surrounding microenvironment [12]. Neurotrophic factors and more recently chemokines have been identified as molecular determinants of PNI, but our knowledge of the biology of tumour cell interactions with nerves remains very poor. Studies have demonstrated that chemokines play an important role in the progression of tumours. Acting as autocrine growth factors, they can accelerate tumour growth via the activation of growth factor receptors [13]. Chemokines also promote the proliferation of tumour cells by making the tumours insensitive to anti-growth signals [14]. Recently, chemokines have been widely investigated in the process of PNI, particularly the CXCR4/CXCL12, CCL2/CCR2, CCL5/CCR5, CXCL13/CXCR5, and CX3CL1/CX3CR1 signalling axes [15]. Importantly, recent studies have demonstrated the reciprocal interactions between other factors (MMPs, NGF, Nuclear factor κB (NF-κB), Slug, Twist) and chemokines in PNI. The expression and roles of chemokines and their receptors in malignant neoplasms can also be regulated by various microenvironmental factors, including chronic inflammation, hypoxia, hepatocyte growth factor (HGF), and vascular endothelial growth factor (VEGF) [12]. Another important category of factors implicated in PNI and in some kinds of tumours proven to modulate the expression of chemokine receptors [16] is the neurotrophin family, a group of small, basic, secreted proteins that aid in the survival and maintenance of specific neuronal populations. This family includes nerve growth factor (NGF), brain-derived neurotrophic factor (BDNF), neurotrophin-3 (NT-3), and neurotrophin-4/5 (NT-4/5). Studies have demonstrated that NT-3 and its specific receptor tropomyosin receptor kinase C (TrkC) are overexpressed in different types of tumours, such as pancreatic and prostate cancer with PNI [17,18]. In addition, emerging evidence has indicated that NT-3 can inhibit myelination by activating TrkC to facilitate the migration of Schwann cells (SCs) [19]. Although knowledge of the role of nerve-cancer crosstalk in tumour progression has been improved by a few landmark discoveries, there is still much to understand about how cancers regulate nerves, as well as the role and mechanism of NT-3 in the progress of PNI.

Support for the clinical application of radiotherapy (RT) in the treatment of cancers with PNI primarily derives from limited retrospective series demonstrating improved local control rates following irradiation of neurotrophic cancers. However, we currently lack a biological understanding of how radiation treatment of PNI translates into improved disease control, and as a consequence, a mechanistic justification for clinical practice is currently unavailable. Primary mucosal melanoma (PMM) and pancreatic ductal adenocarcinoma (PDAC) are aggressive and radioresistant tumours that have shown promising results when treated with carbon ion radiotherapy (CIRT) [20,21]. Currently, C-ions are used to treat deep-seated and radioresistant tumours due to their favourable inverse dose-depth profiles and their higher relative biological effectiveness (RBE) at the end of their range. These special characteristics underlie important benefits for cancer treatment, including higher accuracy, increased efficacy in killing cells, particularly of highly hypoxic regions and radioresistant cellular cycle phase, and the inhibition of angiogenesis [22]. Therefore, CIRT seemed to overcome the intrinsic radioresistance of PMM leading to long-lasting G2/M arrest compared to photon beam radiotherapy (XRT) through the activation of the pRb/E2F1/Chk2 pathway [23]. Despite promising results in terms of local control, the CIRT outcomes have been unrewarding in terms of progression-free survival for both PMM and PDAC [24,25]. One of the challenges is to understand how to overcome the out-of-field progression and metastases after CIRT for both these aggressive malignancies. RT escape might be caused by the presence of micrometastases already present before RT. Several in vitro and in vivo studies have demonstrated that RT can elicit cellular changes that alter the motility of cancer cells. Generally, evidence has indicated that XRT may subsequently enhance the migration and/or invasiveness of cancer cells surviving after irradiation, whereas CIRT diminished these features [26]. Nevertheless, not all cell lines exhibit the same migratory responses following irradiation, as some showed reduced invasiveness after XRT or enhanced invasion after CIRT [27,28]. Because of the complexity of the mechanisms implicated in PNI, little is known about the natural metastatic progression of pancreatic cancer and little more for vaginal PMMs [9]. This study focused on understanding how NT-3 influenced the response to X-RT and CIRT of two radioresistant cell-lines: HMV-II and PANC-1. In particular, the first endpoint of the current research was to investigate the response of vaginal malignant melanoma cells and pancreatic adenocarcinoma cells to NT-3. The secondary endpoint was to evaluate the effects of conventional photon beam radiotherapy (XRT) and CIRT on the cell viability, proliferation, and migration of HMV-II and PANC-1.

## 2. Materials and Methods

### 2.1. Cells and Reagents

The HMV-II (human malignant melanoma) cell line was purchased from Sigma-Aldrich (St. Louis, MO, USA) and the PANC-1 (pancreatic carcinoma) cell line from the Experimental Zooprophylactic Institute of Lombardy and Emilia Romagna. HMV-II cells were cultured in a humidified atmosphere at 37 °C containing 5% CO_2_ with RPMI 1640 medium containing heat-inactivated foetal bovine serum, 100 U/mL penicillin, and 0.1 mg/mL streptomycin. PANC-1 cells were cultured in the same conditions with DMEM medium containing heat-inactivated foetal bovine serum, 100 U/mL penicillin, and 0.1 mg/mL streptomycin. Culture media and all supplements were obtained from Sigma Aldrich. Cells were split using 10% trypsin when confluent.

### 2.2. Irradiations

Exponentially growing HMV-II and PANC-1 cells cultured in T12.5 or T25 culture flasks (Falcon and Corning, respectively) were exposed to 2 Gy or 4 Gy of either photon or C-ion beams. Sham irradiated samples were handled in the same way as the irradiated ones but left on a table in an adjacent room.

XRT was performed with a 6 MV from a LINAC linear accelerator (3 Gy/min as dose rate) in the Radiotherapy Department of the Istituti Clinici Scientifici Maugeri (Pavia, Italy): T25 flasks were filled to the neck with non-complete medium and horizontally positioned above a 1.5 cm-thick layer of Plexiglas, enough to ensure electronic equilibrium with the used beams, which originated from below (180°). Samples were irradiated from the bottom with 2 Gy and 4 Gy.

C-ion irradiations were performed with the fixed horizontal clinical beam-line using the active scanning technique at the National Center for Oncological Hadrontherapy (CNAO) of Pavia (Italy). T12.5 flasks were filled to the neck with non-complete medium and positioned into a water phantom with its entrance window located at the room isocentre. A spread-out Bragg peak (SOBP) was created by modulating 31 beam energies (246–312 MeV/n, equivalent to 120–180 mm, in 2-mm steps of a water-equivalent path length) to generate a 6 cm-thick uniform irradiation region. Cells were placed at a depth of 15 cm, corresponding to the middle of the SOBP. Samples were exposed to 2 Gy and 4 Gy.

Immediately after both types of irradiation, non-complete medium was aspirated and replaced with complete medium for incubation.

### 2.3. Cell Viability

The cell viability of C-ion or photon-irradiated and sham-irradiated HMV-II and PANC-1 cells with the above-reported doses and type of radiation was assessed up to 72 h after irradiation in time intervals of 24 h by means of the trypan blue exclusion method with a LUNA-II TM automated cell counter (Logos Biosystems, Anyang, Republic of Korea). NT-3 at a concentration of 20 ng/mL was added right after exposure [29], and the cells were incubated. At 24, 48 and 72 h following irradiation, cells were detached and centrifuged, and 10 μL of homogeneous cell suspension were mixed with an equal amount of 0.4% trypan blue stain. Ten microlitres of the solution were loaded into the sample chamber of the counting slide for measurements. The viability percentage of the cell suspension was automatically measured by the instrument. Three independent experiments were performed, and average values with standard deviations were determined.

### 2.4. Cell Proliferation

Cell proliferation studies were performed using the OLYMPUS Provi^TM^ CM20 incubation monitoring system, recording the quantitative data on the confluence status of HMV-II and PANC-1 cells seeded into a 12-multiwell, through periodic multiple scans, until reaching 100% of confluence, starting from a 30% of confluence at time point t0. Also in this experiment, the same NT-3 at 20 ng/mL concentration was added right after the exposure and cells were incubated for 24 h before starting to calculate the proliferation. The automated system provided results by determining the average values of three different wells. 

### 2.5. Cell Migration

Cell migration was evaluated through in vitro transwell and scratch migration assays. Concerning the scratch migration assay, non-irradiated and irradiated HMV-II and PANC-1 cells were seeded in a 12-multiwell plate (Corning, Milan, Italy) and incubated until the formation of a confluent monolayer, 24 h after the exposure for both cell lines. Afterwards, a so-called scratch was created using a sterile pipette tip of a 20- to 200-μL micropipette, NT-3 at 20 ng/mL concentration was added to the wells, and both non-irradiated and irradiated cells were incubated. Images of the scratch were obtained every 24 h using a phase-contrast microscope at 40× magnification until closure of the gap. We performed three replications for each condition.

Regarding the transwell migration assay, non-irradiated and irradiated HMV-II and PANC-1 cells were seeded in the upper inserts of a 24-multiwell culture plate (Corning), while in the lower chambers, 20% serum enriched medium was added to attract cells and promote their migration through the 0.8-μm pores of the membrane toward the bottom. NT-3 was added either to the upper inserts or to the lower wells at 20 ng/mL concentration to test its efficacy as an inductor of migration or as chemoattractant for these cells. Twenty-four hours after incubation, the membrane of each insert was cleaned with a cotton-tipped applicator to carefully remove the media and remaining cells that had not migrated to the lower chamber. The migrated cells were fixed with 70% ethanol and stained with May–Grünwald. Once the inserts were dry, they were observed with a phase-contrast microscope at 100× magnification, and images from at least 5 different fields were obtained for every insert corresponding to each condition. We performed at least three replications for each condition.

### 2.6. Statistical Analysis

All data are presented as the means ± standard deviations (SDs) and calculated using GraphPad Prism software, version 8 (GraphPad Software Inc., San Diego, CA, USA). Student’s two-tailed *t*-test was used to evaluate significant differences between two groups. *P*-values ≤ 0.05 were considered to indicate a statistically significant difference. All experiments were replicated at least three times.

## 3. Results

### 3.1. Cell Viability and Proliferation

**Figure 1:**
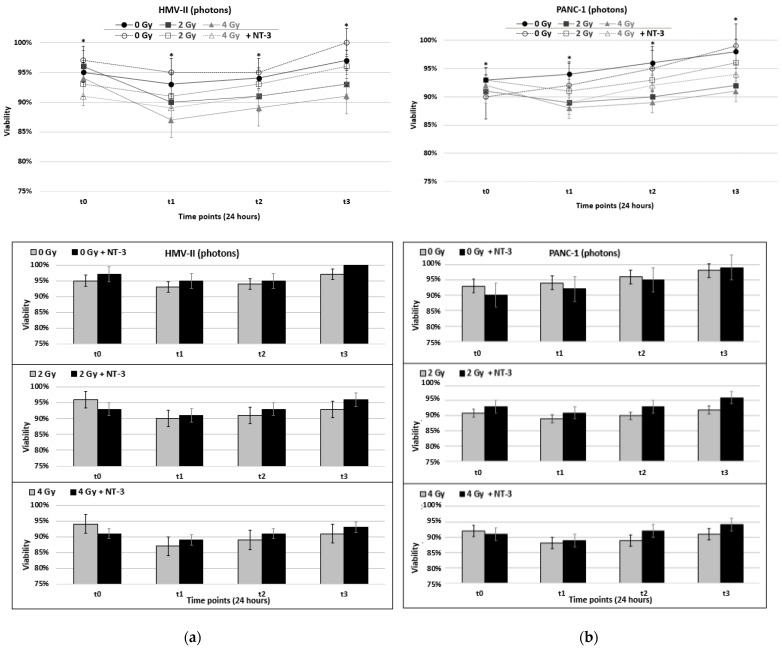

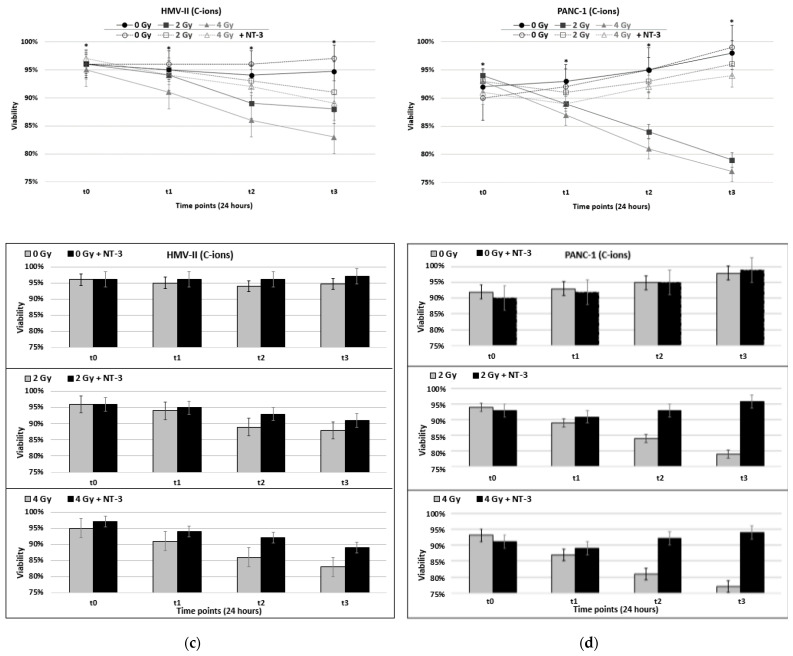
(**a**,**b**) Viability after the exposure of HMV-II (**a**) and PANC-1 (**b**) cells to photons in control conditions and with the addition of NT-3 to each cell line respectively. (**c**,**d**) Viability after the exposure of HMV-II (**c**) and PANC-1 (**d**) cells to C-ions in control conditions and with the addition of NT-3 to each cell line respectively. Bar graphs correspond to each condition represented by the line graphs, separately. * stands for *p* ≤ 0.05 and a statistically significant difference among 0–2 Gy, 0–4 Gy and 0–2 Gy with NT-3, 0–4 Gy with NT-3 for each time point.

After 24 h of exposure, we saw a temporary decrease in the viability values of photon-irradiated PMM and PDAC cells (Figure 1a,b) that already, from the second time point t2 (48 h), started to increase to eventually reach their pre-irradiated baseline levels. For example, 2 Gy of photon-irradiated HMV-II cells at time point t3 reached 93% of viability, a value similar to that of non-irradiated cells. The addition of NT-3 to photon-irradiated HMV-II and PANC-1 cells caused a less marked initial decrease; for photon-irradiated HMV-II cells, indeed, the presence of NT-3 resulted in a 2% decrease in viability at 2 Gy and 4 Gy compared to the control condition, the values of viability of which at the same doses equated to decreases of 6% and 7%, respectively. With regards to PANC-1, the addition of NT-3 after exposure to 2 Gy of photons resulted in a 2%, statistically significant reduction at each time point, whereas at 4 Gy, a 2% decrease persisted until 48 h before becoming 3% at 72 h—differences that were also statistically significant (Figure 1a,b). NT-3 addition induced a further increase in viability levels of non-irradiated cells of both cell lines, following the general trend of each cell line. More specifically, in HMV-II cells, values initially decreased and then increased from time point t2, while in PANC-1 cells, the viability gradually increased.

Moreover, C-ion irradiation without the addition of NT-3 induced a significant decrease in cell viability, measurable already at the first time point of 24 h; in contrast with X-ray, this trend persisted for the whole-time interval observed (Figure 1c,d). Two grays of C-ions led to an 8% decrease and 4 Gy to a 12% statistically significant decrease in viability in HMV-II cells, while 14% and 16% resulted in statistically significant reductions in PANC-1 cells.

C-ion-irradiated cells of both cell lines cultured with NT-3 maintained the same decreasing trend (Figure 1c,d). Compared to photon irradiation, the addition of NT-3 after exposure to C-ions resulted in a smaller (1–2% compared to 4–5% of photons) but statistically significant increase in viability values across all time points.

**Figure 2:**
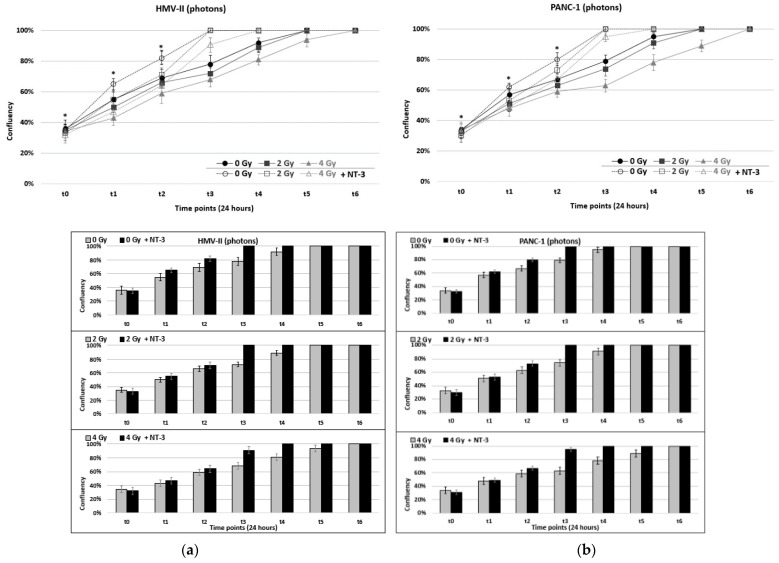

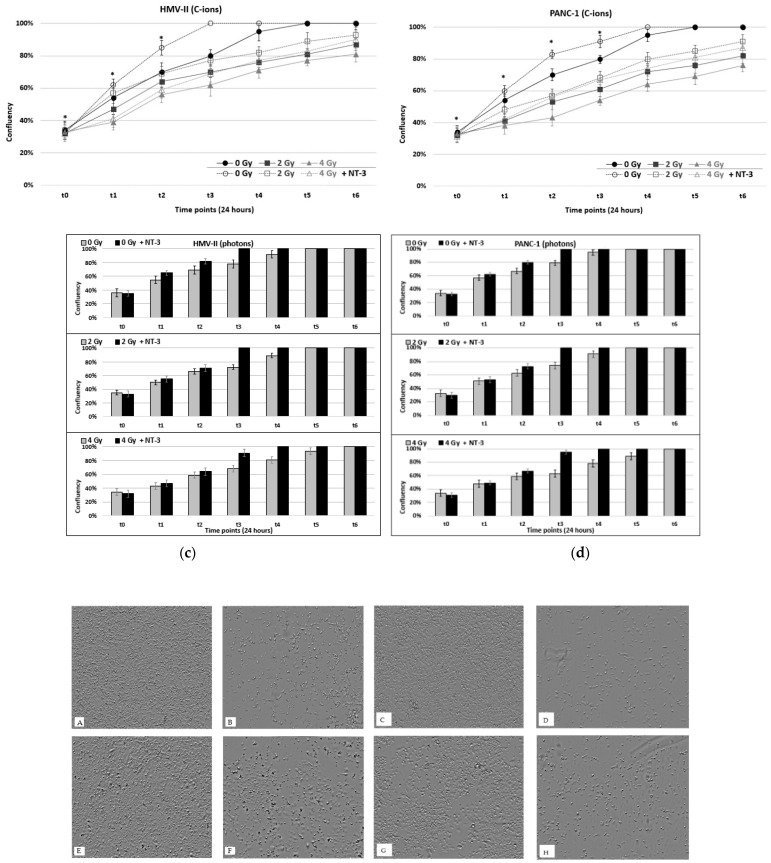
(**a**,**b**) Confluence after the exposure of HMV-II (**a**) and PANC-1 (**b**) cells to photons in control conditions and with the addition of NT-3 to each cell line respectively. (**c**,**d**) Confluence after the exposure of HMV-II (**c**) and PANC-1 (**d**) cells to C-ions in control condition and with the addition of NT-3 to each cell line respectively. Bar graphs correspond to each condition represented by the line graphs, separately. * stands for *p* ≤ 0.05 and statistically significant differences among 0–2 Gy, 0–4 Gy and 0–2 Gy with NT-3, 0–4 Gy with NT-3 for each time point. (**A**–**H**). 4 Gy irradiated HMV-II (**A**,**B**,**E**,**F**) and PANC-1 (**C**,**D**,**G**,**H**) cells with and without the addition of NT-3, respectively, at time point t3 with photons in the left panel and C-ions in the right one. Images obtained with the OLYMPUS Provi^TM^ CM20 incubation monitoring system.

Regarding cell proliferation, the results demonstrated that photon-irradiated cells of both cell lines reached confluence earlier than C-ion-irradiated ones. In particular, the X-ray-treated with 2 Gy HMV-II and PANC-1 cells reached confluence at time point t5 after exposure (Figure 2a,b), but the 4 Gy = irradiated cells of both cell lines reached, respectively, only 81% and 76% confluence at the same time point after CIRT treatment (Figure 2c,d). 

The addition of NT-3 led to earlier confluence in all conditions of both cell lines (i.e., 90% at time point t6 for 4 Gy HMV-II and 87% for 4 Gy PANC-1 C-ion irradiated cells) (Figure 2a–d,A–H).

### 3.2. Cell Migration

**Figure 3:**
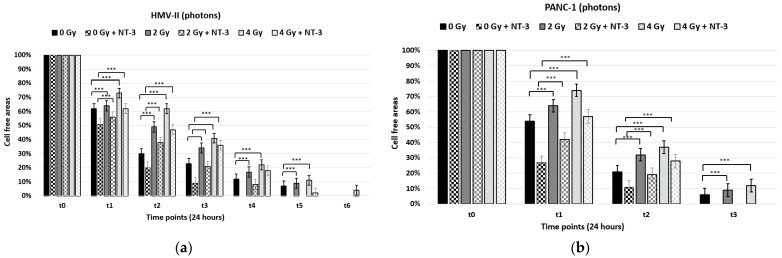
Scratch migration assay after the exposure of HMV-II (**a**) and PANC-1 (**b**) cells to photons without and with the addition of NT-3 to each cell line, respectively. *** stands for *p* ≤ 0.001 and extremely significant statistical differences.

With the scratch migration assay, we observed that the migration capacity of both HMV-II and PANC-1 cells after exposure to photons decreased in a dose-dependent way since the wound closure was slower in irradiated cells compared to control ones and became slower by increasing the dose. Moreover, pancreatic cancer cells presented higher motility after irradiation with photons compared to vaginal mucosal melanoma cells since the wound closed already after time point t3, while it was still open at time point t6 in 4 Gy photon-irradiated HMV-II cells (Figure 3a,b). The addition of NT-3 increased the cell motility of non-irradiated and irradiated cells, and the scratch area closed already at timepoint 5 in HMV-II cells with only a 2% cell-free area in the case of 4 Gy HMV-II cells and after timepoint 2 in PANC-1 cells. However, the general trend of dose-dependence and decrease in migration by increasing the dose persisted.

**Figure 4:**
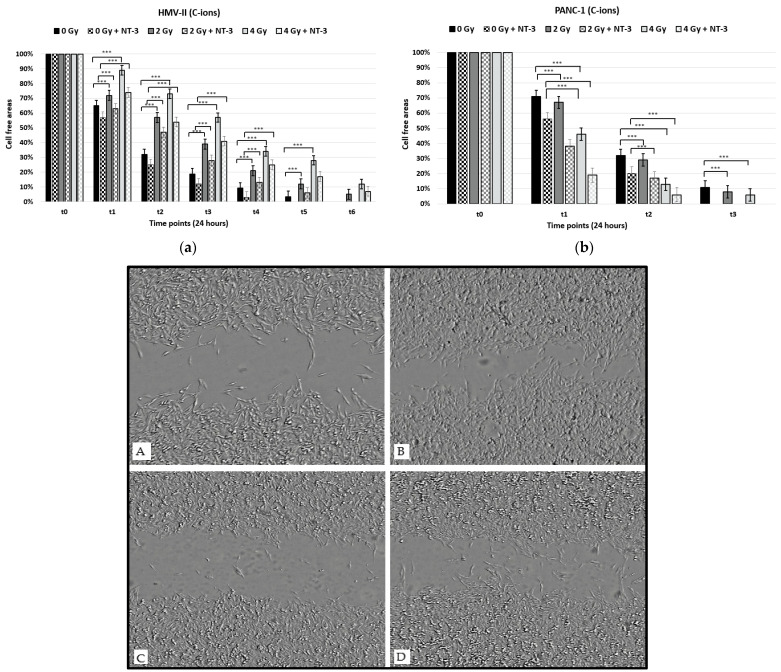
Scratch migration assay after the exposure of HMV-II (**a**) and PANC-1 (**b**) cells to C-ions without and with the addition of NT-3 to each cell line, respectively. *** stands for *p* ≤ 0.001 and extremely significant statistical differences. (**A**–**D**): 4 Gy photon-irradiated and C-ion irradiated HMV-II cells at time point t2 in the control condition (**A**,**C**) and with the addition of NT-3 (**B**,**D**). Images obtained with the OLYMPUS Provi^TM^ CM20 incubation monitoring system.

On the other hand, C-ions cause a more significant decrease in cell migration in vaginal mucosal melanoma cells since cell-free area percentages remain higher in all conditions (i.e., 57% and 34% in C-ion-irradiated cells compared to 41% and 22% in photon-irradiated cells at time point t3 and time point t4, respectively) compared to those irradiated with photons (Figure 4a). Regarding pancreatic cancer cells, hadrontherapy had the opposite effect; C-ions increased cell motility in a dose-dependent manner (Figure 4b). Also in this case, the addition of NT-3 increased the migration capacity of cells, still following the dose-dependence trend for each cell line; a dose-dependent decrease in HMV-II and an increase in PANC-1 cells occurred (Figure 4a,b,A–D).

**Figure 5:**
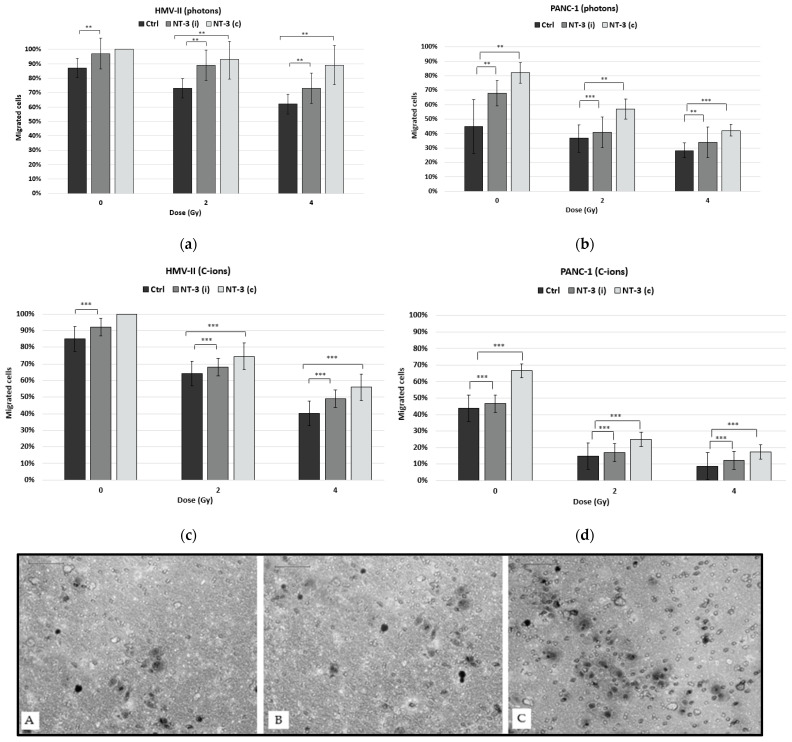
Transwell migration assay after the exposure of HMV-II and PANC-1 cells to photons (**a**,**b**) and C-ions (**c**,**d**). NT-3 was tested both as an inductor [NT-3 (i)] and as a chemoattractant [NT-3 (c)]. ** stands for *p* ≤ 0.01 and highly significant statistical differences, and *** stands for *p* ≤ 0.001 and extremely significant statistical differences. (**A**–**C**): 2 Gy C-ion irradiated PANC-1 cells in control condition (**A**), with NT-3 added as an inductor (**B**), or with NT-3 added as chemoattractant (**C**).

To confirm our results, we also performed the transwell migration assay. Our findings showed that both types of irradiation caused a dose-dependent decrease in cell migration in both cell lines this time (Figure 5a–d). The addition of NT-3, both as an inductor and as a chemoattractant, caused an increase in migration, which was more significant when the neurotrophic factor acted as a chemoattractant (Figure 5A–C).

## 4. Discussion

The central aim of this study was to evaluate the cellular responses of PMM and PDAC in terms of viability, proliferation, and migration after NT-3 conditioning, using low- and high-LET irradiations. Our results demonstrated that both types of irradiation decreased the cell viability and proliferation of the cell lines, even though the cells seemed to repair damage more easily after exposure to photons, reaching higher levels of viability and confluence at an earlier time point when compared to C-ions. This important piece of evidence might justify the different clinical responses of PMM and PDAC when treated with conventional RT or CIRT and may pave the way for new treatment approaches. Indeed, for both PMM and PDAC surgery, when available, irradiation is the cornerstone. However, for gynaecological locally advanced stages or patients with deteriorating health conditions or refusing a demolitive surgical approach, chemotherapy and radiotherapy might be used. Even if preclinical and clinical data have demonstrated that gynaecological melanomas display intrinsic resistance to XRT [24,30], it is sometimes recommended in an adjuvant setting but with unsatisfactory results [31], while CIRT has proved to be effective in unresectable and advanced gynaecological PMM with high rates of local control [24,32,33] and no increased toxicity in cases of combination with immunotherapy [34]. Due to the broad heterogeneity of genetic mutations and dense stromal environment, PDAC is among the most chemoresistant cancers [35]. Even if resection, to date, is considered the only curative treatment for pancreatic cancer, it is limited to only 15–20% of cases with little success since only 20% of resected patients survive more than five years [36]. Post-resection death is often the result of recurrences occurring both locally (33–86%) and distantly (23–92%) [37,38]. Unfortunately, data about radical XRT approaches, although promising, are unsatisfactory, with low rates of local control and dismal overall survival [33]. Even if there were limited data, CIRT seems to be a promising treatment option to improve overall survival and local control in unresectable pancreatic cancer [21]. The better response after CIRT for both cell-lines might support the promising clinical experience of CIRT in these settings.

The main reason for the ineffectiveness of XRT for both PMM and PDAC is the existence of intrinsic and acquired radioresistance, as well as the high capacity for metastatic dissemination to adjacent organs [39,40,41]. In addition to the well-known and above-reported means of metastasis [42], tumour cells can invade both the epineurium and the perineurium and may reach the endoneurium, becoming intimately associated with Schwann cells (SCs) and nerve axons [43]. PNI represents a common histological finding and is considered a marker of poor prognosis for numerous malignant neoplasms, including head and neck, pancreatic, prostate, colorectal, gastric, salivary gland, and breast cancers [44]. PNI is closely associated with increased postoperative locoregional recurrence and a decreased survival rate in several solid tumours, likely because neoplastic cells disseminating along nerve fascicles are spared by macroscopic resection surgery, causing recurrence. Studies have demonstrated that NT-3 can inhibit myelination by activating TrkC to facilitate the migration of SCs [45,46]. Studies have demonstrated that the NT-3/TrkC axis promotes directional migration and inhibits the apoptosis in SCs and SACC cells, thus regulating PNI development and resulting in poor prognosis [29], suggesting that interruption of the interaction between SCs and SACC cells by blocking the NT-3/TrkC axis might be a novel strategy for anti-PNI therapy. To the best of our knowledge, our work investigated for the first time the roles of NT-3 in PMM and PDAC progression after CIRT compared to XRT. To date, there have been only limited retrospective reports supporting the clinical application of radiation for the treatment of cancers exhibiting PNI. Compared with other malignancies, PDAC exhibits one of the highest rates of PNI and is present in about 90% of cases [47]. Extrapancreatic perineural invasion has the strongest prognostic implications and is associated with increased locoregional recurrence, increased distant metastasis, and worse overall survival, and it is present in approximately 86% of patients who have retropancreatic tumour invasion [48]. In 2012, Bakst et al. demonstrated that a single dose of radiation may impair PNI through not only cancer cell death but also independent effects on the nerve itself and the nerve’s production of chemotactic factors [49]. On the other hand, the reported incidence of PNI in melanoma has varied substantially across studies. Although it is well understood that the greatest rates of PNI are seen in the desmoplastic variant, the conflation of PNI with neural transformation in the reporting of other forms of melanoma makes it difficult to obtain a precise figure [50]. In 2018, Zhu et al. treated seven patients suffering from melanoma exhibiting PNI with XRT and demonstrated that this kind of treatment can significantly reduce local recurrences, but not overall survival, since distant metastasis occurs frequently [51].

Regarding migration, photons and C-ions cause a decrease in cell motility that increases after the addition of NT-3 to PMM cells. In contrast, PANC-1 cells exhibit increased cell motility after exposure to C-ions, which could be influenced also by the high proliferative capacity of cells. On the other hand, the transwell migration assay demonstrated that there is a decrease in this motility after exposure to irradiation, which is more significant in the case of C-ions, as well as that the addition of NT-3 caused an increase in this ability, which is higher when the neurotrophic factor acts as a chemoattractant rather than as an inductor of cell migration.

Although knowledge of the role of nerve-cancer crosstalk in tumour progression has been improved by a few landmark discoveries, there is still much to understand about how cancers regulate nerves, as well as the role and mechanism of NT-3 in the progress of PNI in PMM and pancreatic adenocarcinomas.

Our experiments provide evidence that neurotrophic factors accelerate tumour growth and promote proliferation, as well as migration. Thus, hadrontherapy using C-ions could impair the metastatic process by compromising the migration ability of cancer cells since it presents better outcomes compared to the conventional radiotherapy. Additional radiobiological research in this direction may offer important knowledge, leading to better management of PMM and PDAC from a clinical point of view, as well as personalized treatment options for patients suffering from this kind of tumour.

## 5. Conclusions

In conclusion, the results of the in vitro experiments performed with the two cell lines corresponding to neurotropic tumours of PMM and PDAC elucidated the responses of cells to NT-3 following XRT and CIRT treatment. More specifically, our findings demonstrated the ability of the two different protocols of irradiation to decrease cell viability and proliferation in a dose-dependent way, with C-ions exerting more severe effects on these aspects, while their effect on cells’ migration seemed to depend on the cell line and the performed assay. Furthermore, our data highlight that NT-3 was able to promote cell viability, proliferation, and migration, regardless of exposure to irradiation, most likely because of the activation of pathways involved in cell survival and migration. Further studies are required to better understand the mechanisms underlying perineural invasion and NT-3 involvement, as well as the effects of XRT and CIRT, in this metastatic pathway, as well as to improve clinical treatment protocols for patients with such aggressive tumours. Strong collaboration between pre-clinical and clinical research is warranted to take a step forward in the treatment of these difficult-to-cure malignancies.

## Data Availability

Data available on request to the corresponding author.
